# Two validity evidences of the ESQUADA and Brazilians’ dietary quality levels

**DOI:** 10.11606/s1518-8787.2021055002397

**Published:** 2021-08-06

**Authors:** Thanise Sabrina Souza Santos, Pedro Henrique de Moura Araújo, Dalton Francisco de Andrade, Maria Laura da Costa Louzada, Maria Alice Altenburg de Assis, Betzabeth Slater

**Affiliations:** I Universidade de São Paulo Faculdade de Saúde Pública Programa de Pós-Graduação em Nutrição em Saúde Pública São Paulo SP Brasil Universidade de São Paulo. Faculdade de Saúde Pública. Programa de Pós-Graduação em Nutrição em Saúde Pública. São Paulo, SP, Brasil; II Universidade Federal de Santa Catarina Programa de Pós-Graduação em Engenharia de Produção Florianópolis SC Brasil Universidade Federal de Santa Catarina. Programa de Pós-Graduação em Engenharia de Produção, Florianópolis, SC, Brasil; III Universidade Federal de Santa Catarina Departamento de Informática e Estatística Florianópolis SC Brasil Universidade Federal de Santa Catarina. Departamento de Informática e Estatística, Florianópolis, SC, Brasil; IV Universidade de São Paulo Faculdade de Saúde Pública Departamento de Nutrição São Paulo SP Brasil Universidade de São Paulo. Faculdade de Saúde Pública. Departamento de Nutrição. São Paulo, SP, Brasil; V Universidade Federal de Santa Catarina Centro de Ciências da Saúde Florianópolis SC Brasil Universidade Federal de Santa Catarina. Centro de Ciências da Saúde, Florianópolis, SC, Brasil

**Keywords:** Adolescent, Adult, Young Adult, Validation Studies, Surveys and Questionnaires, Food Guide, Diet Surveys

## Abstract

**OBJECTIVE:**

Assess two validity evidences of the diet quality scale (ESQUADA) for the selection of items with better discrimination of the Brazilians’ diet quality and propose a description in score levels.

**METHODS:**

Brazilian adolescents and adults residing in the country (n = 2,059) answered an online questionnaire with 52 items, shared on social networks and email lists between March and April 2018. Statistical tests were applied to analyze the validity and reliability of the instrument’s evidence. Factor analysis was applied to study the dimensionality of the questionnaire items. Item response theory was applied to identify the discrimination and location of items on the continuum, construct the scale and assess the differential item functioning in terms of sex and age.

**RESULTS:**

Among the 52 items of the questionnaire, 25 had greater measurement accuracy, with adequate adjustment and reliability. The item on the habit of eating ultra-processed foods at home showed the best discrimination of diet quality. No item showed differential functioning regarding sex and age. In the construction of the ESQUADA, five diet quality levels were identified: very poor, poor, good, very good and excellent. It was observed that while breakfast cereals and/or cereal bars are more frequently consumed by individuals with “very poor” diet quality; nuts and/or walnuts are most often consumed by those individuals with “excellent” diet quality.

**CONCLUSION:**

The ESQUADA consists of 25 precise items with no differential functioning to assess the quality of Brazilians’ diet. The construction of the ESQUADA made it possible to recognize food consumption and dietary practices characteristic of each level of diet quality.

## INTRODUCTION

Many health problems that affect the world population today are related to unhealthy eating habits. In 2017, inadequate nutrition was the main global risk factor for mortality and the second main factor for years of life lost due to disability^1^ . Its effect on a country’s food system is also observed. In Brazil, the increase in the production and availability of ultra-processed foods has influenced the consumption and health of the population^2^ .

Based on this, in 2014, the Ministry of Health published the second edition of the *Dietary Guidelines for the Brazilian Population* , which brings principles and recommendations for healthy and sustainable eating. The Guideline’s recommendations consider food more than a means of obtaining nutrients, incorporating messages that take into account food processing, combinations of meals, modes of eating, as well as the impact on the environment and the society^3^ .

It is still a challenge to assess and monitor food considering the Guidelines^3^ , due to the lack of instruments. Nutrition has been evaluated by applying diet quality indices based on previous recommendations, which depend on the use of diet surveys and require time for analysis^4^ . Furthermore, these indices are supported by a subjective definition of cutoff points for classification, giving the same importance to items with different impacts on health. Item Response Theory (IRT) is an analysis proposal to overcome this limitation when considering the characteristics of the questionnaire items regarding the ability to discriminate the variable of interest and location in the respective continuum and a probabilistic model to calculate and describe the scores^5^ .

Given this gap, the diet quality scale (ESQUADA) was developed based on the Guidelines’ recommendations^3^ , including, for example, the habit of having main meals and consuming foods such as fruits and sweetened beverages^6^ . The present study sought to assess two validity evidences of the ESQUADA for the selection of items with better discrimination of the diet quality and to propose a description in score levels.

## METHODS

This is a psychometric study to assess the validity of the ESQUADA regarding its internal structure and differential functioning, identifying the final set of items, with IRT’s application.

**Figure 1:**
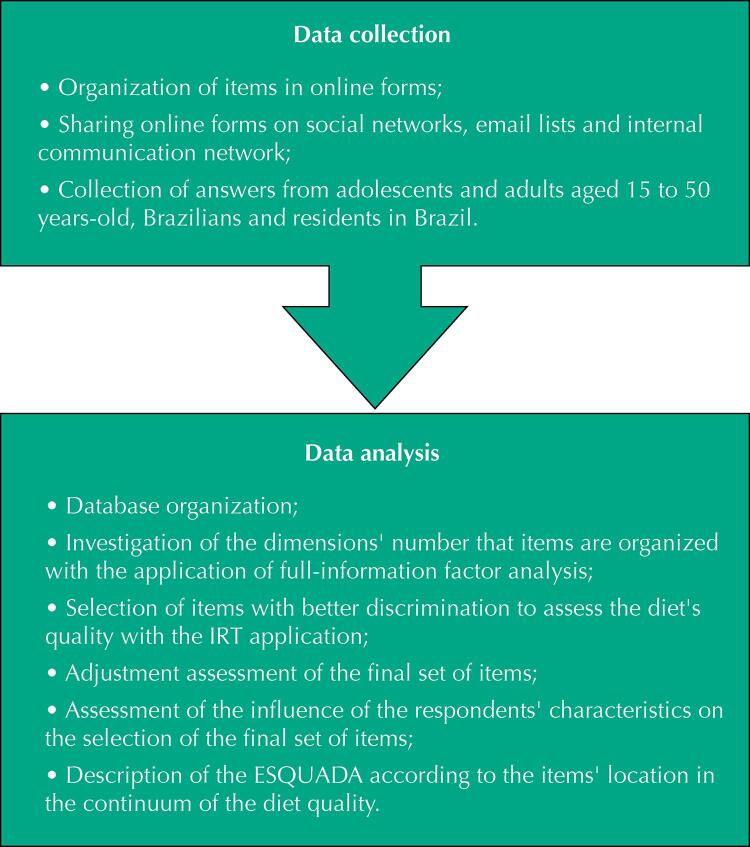
Description of the study phases of two validity evidences of the diet quality scale (ESQUADA).

IRT: item response theory; ESQUADA: diet quality scale.

In a previous study^6^ , experts were asked to assess the items for relevance to measuring the diet’s quality, for consistency with the Guidelines’ recommendations^3^ and for writing’s clarity for adolescents and adults^7^ . After analyzing the experts’ suggestions, the understanding of the items by Brazilian adolescents and adults was assessed^6^ . Subsequently, a total of 52 items were considered for the analysis of the internal structure and differential functioning, presented in this study, which, for better organization, was divided into two phases, described below ( Figure 1 ).

### Data Collection for the Validation Study

The sample of respondents sought to be representative of the latent trait under study^8^ (diet quality), including individuals of different levels.

To minimize boredom when answering the questionnaire, the items were organized into thirteen forms with application of the balanced incomplete block design^6 , 9^ . Each form consisted of sixteen items on diet quality and others on sociodemographic characteristics, such as date of birth, sex, education level and country (of birth and current residence).

The order of presentation of items on the forms was also changed to minimize the influence of position and boredom on the responses’ quality. The forms were entered into the Survey Monkey platform and numbered in ascending order from one to thirteen. The access was made through an electronic address with a programming system to ensure the sequential presentation of the questionnaires, in order to ensure an equal distribution of respondents per form. This electronic address was shared on social networks, email lists and the University of São Paulo’s internal communication network.

During collection, the need to direct the forms’ application to individuals with a certain level of diet quality was evaluated, which is still underrepresented. To meet this representativeness of the latent trait, a complementary collection was made with students from public schools in the state of São Paulo, with the authorization of the State Secretariat, using the computer laboratory and with the presence of a researcher.

Data collection was carried out between March and April 2018, including Brazilian adolescents and adults, aged between 15 and 50, residing in Brazil. All individuals signed a consent term, registering their desire to participate. The study was approved by the Ethics Committee on Human Research of the School of Public Health (protocol number 1,943,099).

Among the individuals who accessed the forms (n = 2,373), those who did not meet the inclusion criteria (n = 270), did not answer or answered only one of the items on diet quality (n = 11) and those who accessed the forms more than once (n = 33) were excluded. The final sample included 2,059 individuals.

#### Data Analysis for the Scale Construction

The categories of responses were numbered in ascending order from number one, consistent with the cumulative characteristic of the latent trait. In this sense, number 1 identified the answer option whose habit suggests a poorer diet quality. For example, the item about replacing snacks for lunch or dinner had five response options: “I am not used to”, “Yes, sometimes”, “Yes, I usually replace it one or two days a week”, “Yes, I usually replace it three or more days a week” e “Yes, I usually replace it five or more days a week”. While the highest frequency of replacement, which suggests poorer diet quality, was identified as number one; the unusual was identified as number five.

#### Analysis of the internal structure of the item set

The study of dimensionality was carried out with full-information factor analysis, considering unidimensionality when the first factor explains 20% or more of the variance^10^ . Items with commonality less than 0.20 and factor loading less than 0.30^11^ were excluded from the following analyses.

Next, the probability of individuals choosing category k for each item was calculated using the Samejima graded response model, represented by equation (1)^12^ :


$$P_{i,k}\left(\theta_{j}\right)=\frac{1}{1+\exp^{-a_{i}\left(\theta_{j}-b_{i,k}\right)}}-\frac{1}{1+\exp^{-a_{i}\left(\theta_{j}-b_{i,k+1}\right)}}$$ (1)

Where:

θ_j_ – IRT score for individual *j* ;

P_i,k_(θ_j_) – probability of individual *j* choosing category *k* when answering item *i* ;

a_i_ – item *i* discrimination parameter;

b_i,k_ – location parameter of category *k* of item *i* , with b_i,2_ ≤ b_i,3_ ≤ b_i,4_ ≤ ….

The discrimination parameter (a_i_) indicates the ability of each item to discriminate individuals with different levels of the latent trait. Items with higher values of this parameter show better discrimination of the latent trait^5^ . Items with low discrimination, that is, with a_i_ parameter equal to or less than 0.70, were excluded from the analyses^8^ .

The location parameter (b_i,k_) identifies the location of each item’s response categories across the continuum of the latent trait^5^ . Items with estimates of b_i,k_ with high standard errors, when compared to other items^13^ , and with overlap in the item characteristic curve (ICC), the graphical representation of the model above, were recategorized. Those items that maintained high standard error values even after recategorization were excluded from the analyses.

The adjustment of the model was evaluated by the root mean square error of approximation (RMSEA), standardized root mean square residual (SRMSR), comparative fit index (CFI) e Tucker–Lewis index (TLI)^14 , 15^ . The goodness-of-fit is confirmed when RMSEA and SRMSR values are less than or equal to 0.05 and CFI and TLI values are greater than 0.90^16^ . The accuracy of the ESQUADA was also analyzed using the test information curve (TIC), which represents the sum of the information on the items that compose it. The study of empirical reliability, a measure analogous to Cronbach’s α, allowed us to identify the reliability of the ESQUADA, accepted when reached a value greater than 0.70^11^ .

#### Analysis of consequence of use

Considering that age and sex can influence the probability of individual *j* choosing category *k* for item *i* , the differential item functioning (DIF) was evaluated according to these variables. A p-value less than 0.05 was adopted as the level of significance. DIF detection was also evaluated in the TIC analysis^17^ .

#### Construction of the scale

The probability of choosing category *k* was calculated for each item at different diet quality scores. The item was placed in the score where this probability was greater than or equal to 0.5. Items with better discrimination of diet quality (a_i_ ≥ 1.00) were considered anchors for the description of dietary practices and consumption of foods characteristic of each score. The scores were grouped into levels indicative of the cumulative characteristic of diet quality, allowing to identify that the higher the score, the lower the frequency of replacing meals with snacks, for example. This step was carried out by four nutritionists (TSSS, MLCL, MAAA and BSV). For better interpretability of the ESQUADA^5^ , the scores estimated on a scale with a mean equal to 0 and a standard deviation equal to 1 were later transformed to a scale with a mean equal to 250 and a standard deviation equal to 50.

Analyses were performed using the R software (crossdes and mirt packages) and Microsoft Excel version 2013.

## RESULTS

Respondents represented the variability of the latent trait in order to guarantee the estimation of the parameters a_i_ and b_i,k_. Most of these respondents were female (70%), had no higher education (58%) and lived in the Southeast region of Brazil (79%). Filling out the forms took between six and eight minutes.

The 52 items (I-01 to I-52) considered for the analysis explained 0.20 of the variance, but 15 of them were excluded due to the low values of commonality, factor loading and discrimination parameter (I-06, I-17, I-18, I-19, I-20, I-23, I-26, I-27, I-28, I-30, I-33, I-34, I-38, I-45 and I-49). Analyses were repeated without these items (n = 37), recategorizing those with estimates with high standard error values for the parameter b_i,k_ and overlap in the ICC. In the subsequent analyses, 12 of these items had low values of commonality, factor loading and discrimination parameter and were excluded (I-05, I-07, I-10, I-12, I-14, I-15, I-16, I-29, I-35, I-37, I-39 and I-47).

**Figure 2:**
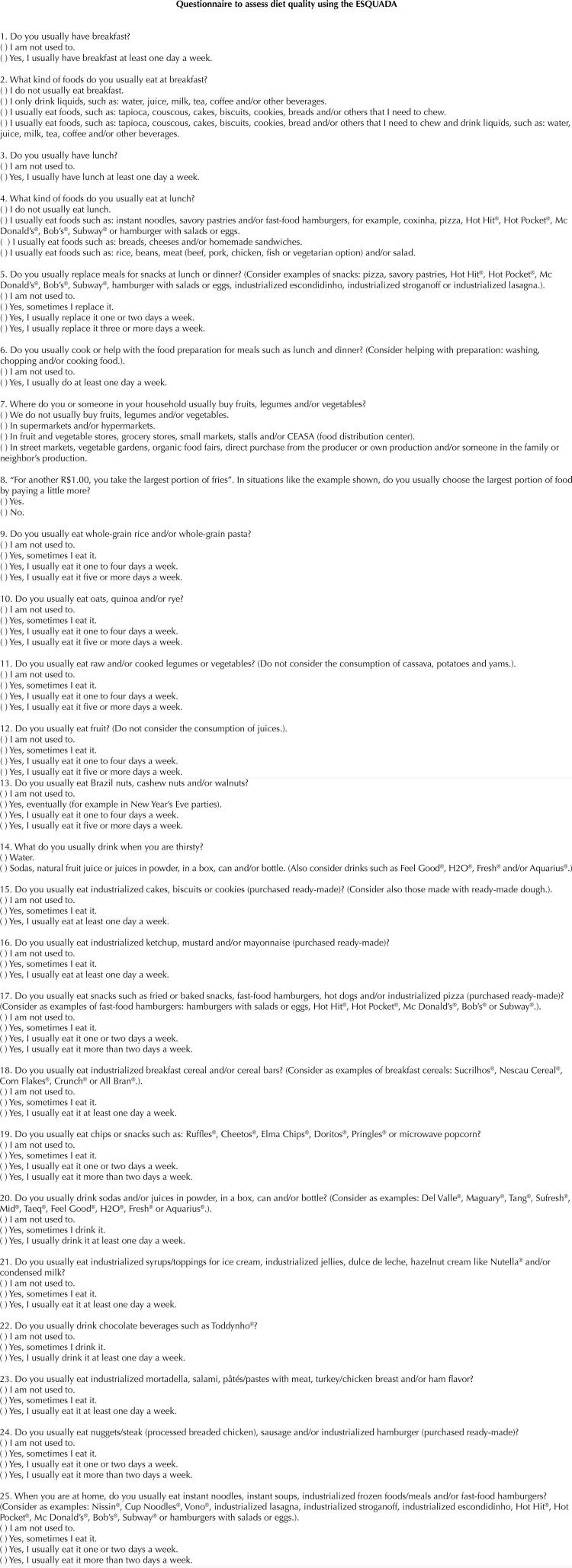
Questionnaire with items and answer options that make up the diet quality scale (ESQUADA).

The remaining 25 items presented adequate values for the parameter a_i_ and b_i,k_ and respective standard errors, explaining 0.34 of the variance. The values of commonality, factor loading and parameters a_i_ and b_i,k_ are described in Table 1 . The next results focus on the 25 items that remained in the final set, making up the ESQUADA. Items and response options are shown in Figure 2 .

**Table 1:** Description of commonality, factor loading and discrimination and location parameters values and respective standard errors of each item of the diet quality scale (ESQUADA) (n = 2,059).

Items	Communality	Factor Loading	a_i_ (SE)	b_i,2_ (SE)	b_i,3_ (SE)	b_i,4_ (SE)
I-01-Do you usually have breakfast?	0.364	0.603	1.288 (0.154)	-0.637 (0.090)		
I-02-What kind of foods do you usually eat at breakfast?	0.241	0.491	0.959 (0.125)	-1.673 (0.198)	-0.108 (0.098)	
I-03-Do you usually have lunch?	0.264	0.514	1.021 (0.202)	-2.750 (0.427)		
I-04-What kind of foods do you usually eat at lunch?	0.372	0.610	1.309 (0.343)	-3.457 (0.662)		
I-08-Do you usually replace meals for snacks at lunch or dinner? (Consider examples of snacks: pizza, savory pastries, Hot Hit^®^, Hot Pocket^®^, Mc Donald’s^®^, Bob’s^®^, Subway^®^, hamburger with salads or eggs, industrialized escondidinho, industrialized stroganoff or industrialized lasagna.).	0.264	0.514	1.020 (0.114)	-3.429 (0.356)	-1.937 (0.195)	0.521 (0.101)
I-09-Do you usually cook or help with the food preparation for meals such as lunch and dinner? (Consider helping with preparation: washing, chopping and/or cooking food.).	0.206	0.454	0.868 (0.132)	-0.245 (0.111)		
I-11-Where do you or someone in your household usually buy fruits, legumes and/or vegetables?	0.176	0.419	0.786 (0.107)	0.304 (0.117)	2.305 (0.294)	
I-13- “For another R$1.00, you take the largest portion of fries”. In situations like the example shown, do you usually choose the largest portion of food by paying a little more?	0.239	0.489	0.954 (0.127)	0.457 (0.108)		
I-21-Do you usually eat whole-grain rice and/or whole-grain pasta?	0.261	0.511	1.011 (0.110)	-0.029 (0.095)	1.278 (0.145)	2.932 (0.298)
I-22-Do you usually eat oats, quinoa and/or rye?	0.476	0.690	1.624 (0.163)	-0.027 (0.068)	1.291 (0.106)	2.494 (0.196)
I-24-Do you usually eat raw and/or cooked legumes or vegetables? (Do not consider the consumption of cassava, potatoes and yams.).	0.414	0.643	1.429 (0.147)	-2.069 (0.176)	-0.771 (0.089)	0.599 (0.087)
I-25-Do you usually eat fruit? (Do not consider the consumption of juices.).	0.295	0.544	1.102 (0.118)	-2.622 (0.254)	-0.768 (0.105)	0.888 (0.117)
I-31-Do you usually eat Brazil nuts, cashew nuts and/or walnuts?	0.362	0.601	1.281 (0.139)	-0.409 (0.084)	1.075 (0.114)	3.080 (0.291)
I-32-What do you usually drink when you are thirsty?	0.200	0.448	0.852 (0.171)	-2.743 (0.475)		
I-36-Do you usually eat industrialized cakes, biscuits or cookies (purchased ready-made)? (Consider also those made with ready-made dough.).	0.276	0.525	1.050 (0.117)	-0.863 (0.118)	1.601 (0.164)	
I-40-Do you usually eat industrialized ketchup, mustard and/or mayonnaise (purchased ready-made)?	0.199	0.446	0.849 (0.117)	-2.249 (0.286)	1.759 (0.236)	
I-41-Do you usually eat snacks such as fried or baked snacks, fast-food hamburgers, hot dogs and/or industrialized pizza (purchased ready-made)? (Consider as examples of fast-food hamburgers: hamburgers with salads or eggs, Hot Hit^®^, Hot Pocket^®^, Mc Donald’s^®^, Bob’s^®^ or Subway^®^.).	0.429	0.655	1.476 (0.153)	-2.853 (0.248)	-1.286 (0.111)	1.339 (0.118)
I-42-Do you usually eat industrialized breakfast cereal and/or cereal bars? (Consider as examples of breakfast cereals: Sucrilhos^®^, Nescau Cereal^®^, Corn Flakes^®^, Crunch^®^ or All Bran^®^.).	0.165	0.407	0.758 (0.127)	-3.738 (0.582)	-0.824 (0.164)	
I-43- Do you usually eat chips or snacks such as: Ruffles^®^, Cheetos^®^, Elma Chips^®^, Doritos^®^, Pringles^®^ or microwave popcorn?	0.448	0.670	1.535 (0.158)	-3.117 (0.287)	-1.979 (0.164)	0.439 (0.073)
I-44-Do you usually drink sodas and/or juices in powder, in a box, can and/or bottle? (Consider as examples: Del Valle^®^, Maguary^®^, Tang^®^, Sufresh^®^, Mid^®^, Taeq^®^, Feel Good^®^, H2O^®^, Fresh^®^ or Aquarius^®^.).	0.514	0.717	1.749 (0.162)	-0.430 (0.071)	0.863 (0.084)	
I-46-Do you usually eat industrialized syrups/toppings for ice cream, industrialized jellies, dulce de leche, hazelnut cream like Nutella^®^ and/or condensed milk?	0.317	0.563	1.160 (0.133)	-1.891 (0.188)	1.118 (0.133)	
I-48-Do you usually drink chocolate beverages such as Toddynho^®^?	0.494	0.703	1.682 (0.187)	-1.608 (0.133)	-0.156 (0.068)	
I-50-Do you usually eat industrialized mortadella, salami, pâtés/pastes with meat, turkey/chicken breast and/or ham flavor?	0.344	0.586	1.231 (0.123)	-1.607 (0.149)	0.906 (0.107)	
I-51-Do you usually eat nuggets/steak (processed breaded chicken), sausage and/or industrialized hamburger (purchased ready-made)?	0.558	0.747	1.912 (0.199)	-2.471 (0.191)	-1.560 (0.113)	0.319 (0.066)
I-52-When you are at home, do you usually eat instant noodles, instant soups, industrialized frozen foods/meals and/or fast-food hamburgers? (Consider as examples: Nissin^®^, Cup Noodles^®^, Vono^®^, industrialized lasagna, industrialized stroganoff, industrialized escondidinho, Hot Hit^®^, Hot Pocket^®^, Mc Donald’s^®^, Bob’s^®^, Subway^®^ or hamburgers with salads or eggs.).	0.633	0.796	2.237 (0.212)	-2.292 (0.158)	-1.473 (0.097)	0.134 (0.060)

SE: standard error; a_i_: item *i* discrimination parameter; b_i,2_: category 2 location parameter of item *i* ; b_i,3_: category 3 location parameter of item *i* ; b_i,4_: category 4 location parameter of item *i* .

This is a free translation of the items from ESQUADA, that is, without following a cross-cultural adaptation and validation process.

The item on the places where individuals usually buy fruits and vegetables showed the lowest discrimination of diet quality (a_11_ = 0.786). On the other hand, the item on the habit of eating ultra-processed foods at home had the best discrimination (a_52_ = 2.237).

The item on the habit of eating breakfast cereals and/or cereal bars had the lowest value for the location parameter (b_42,2_ = -3.738), suggesting that these foods are more frequently consumed by individuals with lower levels of diet quality. On the other hand, the items on the habit of eating nuts and/or walnuts (b_31,4_ = 3.080) and whole-grain rice/pasta (b_21,4_ = 2.932) had the highest values of b_i,k_, suggesting that these foods are consumed more frequently by individuals with higher levels of diet quality.

**Figure 3:**
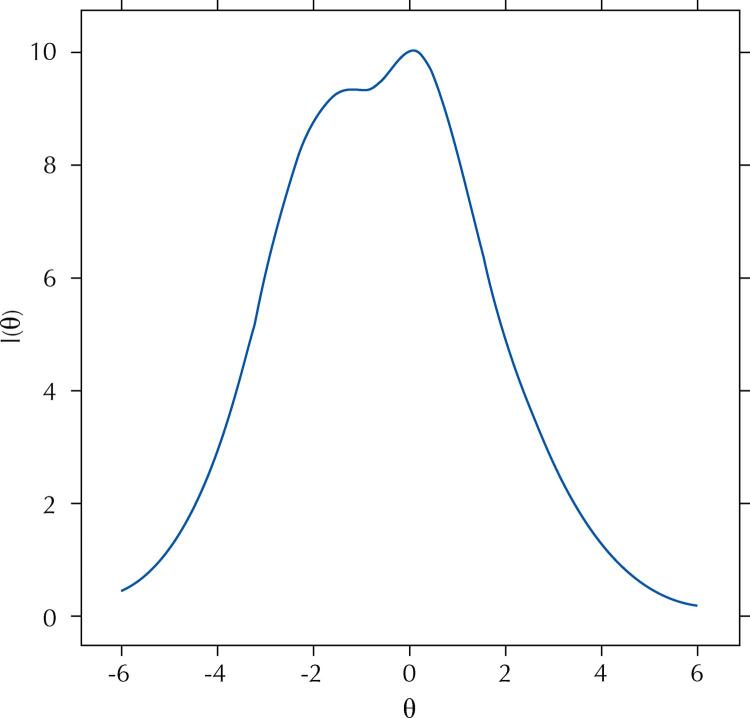
Diet quality scale (ESQUADA) information curve (n = 2,059).

ESQUADA presented an excellent goodness-of-fit (RMSEA = 0.01; SRMSR = 0.02; CIF = 0.99 and TLI = 0.99), with better accuracy between the -2 and +2 scores ( Figure 3 ) and an adequate empirical reliability (0.70). Differential functioning was not identified for any item, considering both age and sex.

θ: score; I(θ): score information.

**Figure 4:**
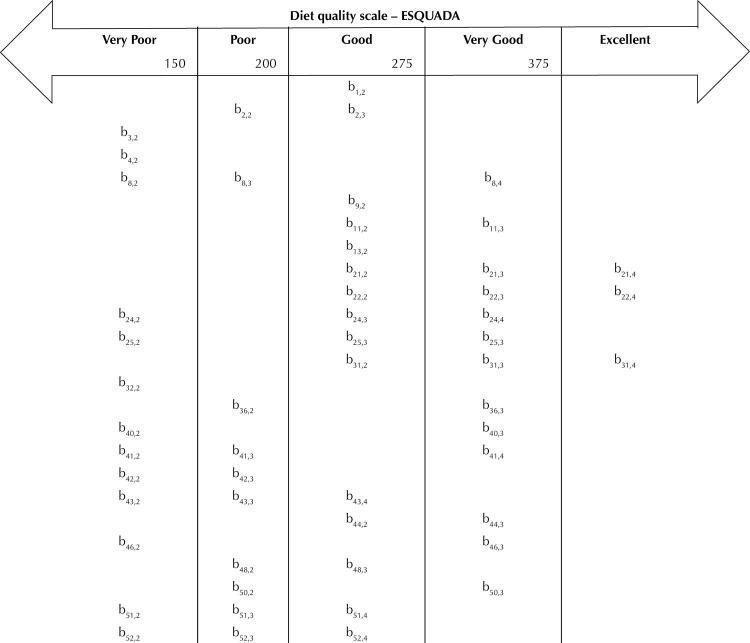
Positioning of items and respective response options in the five levels of the diet quality scale (ESQUADA).

For better interpretability of the ESQUADA, the transformation of the scores for a scale with a mean equal to 250 and a standard deviation equal to 50 was obtained by the constant: θ_(250,50)_ = 59.09 × θ_(0,1)_ + 250.12. The scores were grouped to present the five interpretation levels of the ESQUADA: “very poor”, “poor”, “good”, “very good” and “excellent” ( Chart 1 ). The items and respective answer options that make up each level are shown in Figure 4 .

**Chart 1:** Brief description of the levels of the diet quality scale (ESQUADA).

Classification	Scale (0,1)	Scale (250,50)	Brief description
Very Poor	Less than or equal to -2	Less than or equal to 150	Individuals consume ultra-processed foods and replace meals for snacks on one or two days a week. Individuals consume fruits and vegetables on less than one day a week.
Poor	Greater than -2 and less than or equal to -1	Greater than 150 and less than or equal to 200	Individuals maintain their consumption of fruits and vegetables on less than one day a week. Individuals consume ultra-processed foods and replace meals for snacks on less than one day a week.
Good	Greater than -1 and less than or equal to 0.5	Greater than 200 and less than or equal to 275	Individuals maintain replacing meals for snacks on less than one day a week. Individuals do not consume some ultra-processed foods but consume sugary beverages on less than one day a week. Individuals consume fruits, legumes and vegetables on one to four days a week and oats, rye, quinoa, nuts, walnuts and whole-grain rice/past on less than one day a week. Individuals eat breakfast at least one day a week.
Very Good	Greater than 0.5 and less than or equal to 2.5	Greater than 275 and less than or equal to 375	Individuals do not replace meals for snacks. Individuals do not consume any ultra-processed foods. Individuals maintain eating breakfast on at least one day a week. Individuals consume fruits, legumes and vegetables on five or more days a week and oats, rye, quinoa, nuts, walnuts and whole-grain rice/pasta on one to four days a week.
Excellent	Greater than 2.5	Greater than 375	Individuals maintain not replacing meals for snacks and not consuming ultra-processed foods. Individuals maintain eating breakfast at least one day a week and consume fruits, legumes and vegetables on five or more days a week. Individuals consume oats, rye, quinoa, nuts, walnuts and whole-grain rice/pasta on five or more days a week.

b_i,k_: category *k* location parameter of item *i* .

## DISCUSSION

In this study, two validity evidences of the ESQUADA, a diet quality scale that includes consumption of natural and minimally processed and ultra-processed foods and dietary practices (such as eating breakfast, cooking and replacing meals with snacks), were evaluated. The IRT usage allowed the selection of items with better diet quality discrimination and the results of the analysis of the goodness-of-fit and reliability support the accuracy of the measure presented by the ESQUADA. The TIC allowed us to visualize the measurement error in the entire scale, suggesting that the ESQUADA presents better accuracy between the -2 and +2 scores. In addition, the differential item functioning analysis indicated that the measure is valid in different samples. In other words, the ESQUADA measures with the same precision the diet quality of individuals regardless of sex and age. The absence of DIF highlights the previous study regarding the clarity of the items for Brazilian adolescents and adults^6^ . The analyses also allowed the proposal to describe dietary practices and food consumption at five levels.

After the Guidelines publication^3^ , three aspects were highlighted: (1) the understanding that foods are not just sources of nutrients; (2) the importance of considering not only food consumption but also dietary practices; and (3) the methodological limitations of the statistics used in the development of diet quality indices. ESQUADA aims to assess the diet quality in accordance with the Guidelines recommendations^3^ , considering other dietary characteristics than just the nutritional factor, with the application of more robust statistics. ESQUADA presents itself, then, as the first scale that assesses the diet quality in accordance with the Guidelines^3^ , with the application of the IRT, an analysis centered on the items and not just on the score.

Studies in the field of Nutrition express in a hegemonic way the biomedical paradigm according to the logic of natural sciences, limiting the understanding of the multiple ways in which food interferes in people’s lives. The meaning of eating has been undergoing changes, that is, from a reductionist concept, in the sense of an exclusive supply of energy and nutrients, it expresses the experiences of human beings, marked by nature and culture. In this sense, eating is a natural act, which is expressed in the most cultural ways: in the modes of eating and preparing food, in food choices and preferences, in commensality and in all transformations from food to consumption^20 , 21^ .

In ESQUADA, the habit of cooking at least one day a week, assessed by I-09, identified a better diet quality, indicating the care taken in reserving some time to cook during the week, even with the intense work routine. Considering that the population is cooking at home less frequently^22^ , although one day a week is still insufficient, the item is able to discriminate the diet quality.

Items on the consumption of vegetables (I-24) and fruit (I-25) and place of purchase of these foods (I-11) adhered to the criteria for maintenance in the final set. The consumption of vegetables is an important point for healthy eating and a global strategy for the prevention and control of chronic noncommunicable diseases^23^ . The Guidelines recommends preferring the consumption of fruits to drinking natural juice, as well as buying food in places where there is greater availability of natural food^3^ . The place where the population supplies itself with fruits and vegetables can also indicate the diet quality. In Brazil, purchases at street markets, small markets or directly from the producer were related to lower consumption of ultra-processed foods^24^ .

One of the ESQUADA’s items (I-32) sought to investigate the beverage ingested by individuals when they are thirsty: water, natural fruit juice or sweetened beverages. It presented an adequate discrimination of diet quality, with low values for the location parameter, that is: individuals with low diet quality scores drink water when they are thirsty. In accordance with the recommendations for diet quality indicators^25^ , ESQUADA does not quantify water intake in milliliters or cups but assesses a habit that is directly related to healthy eating and inversely to the obesity incidence^26^ . The presence of this item in the ESQUADA highlights its importance in assessing the diet quality.

Scientific evidence points to the importance of breakfast for nutrition and health^27^ . ESQUADA’s description ( Chart 1 ) indicates that the diet quality increases as individuals adopt the habit of eating breakfast, lunch and not replacing meals with snacks. It must be considered that food intake is a complex behavior. For example, at a “very poor” level of diet quality, individuals tend to eat a traditional meal (with rice, beans, meat or a vegetarian option and/or salad) for lunch and substitute meals for snacks and consume ultra-processed foods. On the other hand, the habit of eating breakfast was only observed from the level of “good” diet quality, in which there is a lower frequency of substitution of meals for snacks and consumption of ultra-processed foods. In other words, the habit of eating breakfast is present at a level of diet quality in which individuals tend to have lunch and consume ultra-processed products less frequently.

Among the 25 items in the ESQUADA, 11 assess the consumption of ultra-processed foods. The item that investigates the consumption of breakfast cereals and cereal bars (I-42) had the lowest value for the location parameter (b_42,2_ = -3.738), suggesting that these foods are frequently consumed at the lowest levels of diet quality. Frequent consumption of ultra-processed foods was associated with a worse nutritional profile in adolescents and adults^28^ . This consumption has been stimulated by marketing strategies, such as the use of terms on product labels, for example: traditional and homemade^29^ . Contemplating some of this influence, ESQUADA includes an item that assesses the habit of choosing the largest portion of foods when the price difference is small in relation to the smallest portion (I-13).

In the description of the ESQUADA ( Chart 1 ), it is observed that individuals stop consuming some ultra-processed foods at the same level of diet quality as they start consuming whole-grain cereals (I-21, I-22) and oilseeds (I-31). Ultra-processed foods only cease to be part of the diet at the last two levels of the ESQUADA, when it is also observed that natural and minimally processed foods are incorporated into the diet. These results reinforce the complexity of assessing diet quality. Thus, individuals with median scores, that is, with a “good” diet quality, present healthy and unhealthy eating habits at the same time. Given this complexity, the application of IRT allowed to identify the items that best discriminate the diet quality^5^ .

Therefore, the ESQUADA is presented as a new measure to accurately assess the diet quality in future studies. To this end, the discrimination and location parameters and the transformation constant presented here should be considered in the next applications of the ESQUADA. With the equalization technique^5^ , researchers will be able to include other items in the measurement, estimating their parameters based on the discrimination and location of those that make up the ESQUADA and expanding the assessment of the diet quality. Additionally, the ESQUADA is easy to apply and analyze and, with its reapplication, it is possible to assess the scores of the same group of individuals, for example, after an intervention in health and nutrition.

Some positive aspects and limitations of this study should be pointed out. Even though the ESQUADA assesses different aspects of diet quality, the final set of items is more related to issues related to food consumption. Furthermore, the analyses indicated the need to recategorize some answer options due to the smaller number of individuals who selected them as answers. However, the number of respondents was sufficient to obtain adequate estimates of the item’s parameters, as well as accurate information on the diet quality. The study features highlights: ESQUADA is based on current recommendations for the nutrition of the Brazilian population and in more robust analyses for selection of items with better discrimination of diet quality, allowing to locate and describe consumption and food practices in its continuum.

## CONCLUSION

The ESQUADA consists of 25 items that assess food consumption and dietary practices in accordance with the Guidelines. The exclusions do not contradict current recommendations, allowing the selection of the most accurate items to assess the diet quality in future studies and contribute to the state of the art in nutritional epidemiology.

In this sense, items on the habit of cooking, eating breakfast, where individuals buy fruits and vegetables, and consumption of natural and ultra-processed foods exemplify the final set that makes up the ESQUADA. The analyses also provided a breakdown of food consumption and dietary practices characteristic of each level of diet quality. In order to complement the study of the two validity evidences of the ESQUADA presented, future articles will be able to investigate the associations between the scores and other measures related to health and nutrition.
