# Erratum

**DOI:** 10.11606/s1518-8787.2023057002397err

**Published:** 2023-03-15

In the article “ **Two validity evidences of the ESQUADA and Brazilians’ dietary quality levels** ”, DOI https://doi.org/10.11606/s1518-8787.2021055002397, published on the Revista de Saúde Pública.2021;55:39, on page 10, the RSP corrects an excerpt in the last paragraph of the results section.

**Where it reads:**

θ_(250.50)_ = 59.09 × θ_(0.1)_ + 250.12

**It should read:**

θ_(250.50)_ = 50 × θ_(0.1)_ + 250.12

