# Supplementary material for: Two validity evidences of the ESQUADA and Brazilians’ dietary quality levels
Source: Rev Saude Publica. 2021 Aug 6;55:39. doi: 10.11606/s1518-8787.2021055002397 (PMC8352565; doi:10.11606/s1518-8787.2021055002397)
Supplement: Translation

# Translation: Duas evidências de validade da ESQUADA e níveis de qualidade da dieta dos brasileiros

**Authors:** Thanise Sabrina Souza Santos, Pedro Henrique de Moura Araújo, Dalton Francisco de Andrade, Maria Laura da Costa Louzada, Maria Alice Altenburg de Assis, Betzabeth Slater

**Affiliations:** I Brasil Universidade de São Paulo. Faculdade de Saúde Pública. Programa de Pós-Graduação em Nutrição em Saúde Pública. São Paulo, SP, Brasil; II Brasil Universidade Federal de Santa Catarina. Programa de Pós-Graduação em Engenharia de Produção, Florianópolis, SC, Brasil; III Brasil Universidade Federal de Santa Catarina. Departamento de Informática e Estatística, Florianópolis, SC, Brasil; IV Brasil Universidade de São Paulo. Faculdade de Saúde Pública. Departamento de Nutrição. São Paulo, SP, Brasil; V Brasil Universidade Federal de Santa Catarina. Centro de Ciências da Saúde, Florianópolis, SC, Brasil

**Keywords:** Adolescente, Adulto, Adulto de Meia Idade, Estudos de Validação, Inquéritos e Questionários, Guias Alimentares, Inquéritos sobre Dietas

## Abstract

**OBJETIVO:**

Avaliar duas evidências de validade da escala de qualidade da dieta (ESQUADA) para seleção dos itens com melhor discriminação da qualidade da dieta dos brasileiros e propor uma descrição em níveis de escore.

**MÉTODOS:**

Adolescentes e adultos brasileiros e residentes no país (n = 2.059), responderam a um questionário on-line com 52 itens, compartilhado em redes sociais e listas de correio eletrônico entre março e abril de 2018. Foram aplicados testes estatísticos para análise de evidências de validade e confiabilidade do instrumento. A análise fatorial foi aplicada para estudo da dimensionalidade dos itens do questionário. A teoria de resposta ao item foi aplicada para identificar a discriminação e localização dos itens no *continuum* , construir a escala e avaliar o comportamento diferencial dos itens quanto ao sexo e idade.

**RESULTADOS:**

Dentre os 52 itens do questionário, 25 apresentaram maior precisão de medida, com ajuste e confiabilidade adequados. O item sobre o costume de comer alimentos ultraprocessados em casa apresentou a melhor discriminação da qualidade da dieta. Nenhum item apresentou comportamento diferencial quanto a sexo e idade. Na construção da ESQUADA foram identificados cinco níveis de qualidade da dieta: “muito ruim”, “ruim”, “boa”, “muito boa” e “excelente”. Observou-se que enquanto cereais matinais e/ou barrinhas de cereais são consumidos com maior frequência por indivíduos com qualidade da dieta “muito ruim”; castanhas e/ou nozes são consumidos mais frequentemente por aqueles indivíduos com qualidade da dieta “excelente”.

**CONCLUSÕES:**

A ESQUADA é composta por 25 itens precisos e sem comportamento diferencial para avaliar a qualidade da dieta dos brasileiros. A construção da ESQUADA possibilitou reconhecer consumo e práticas alimentares característicos de cada nível de qualidade da dieta.

## INTRODUÇÃO

Muitos problemas de saúde que acometem a população mundial na atualidade se relacionam a práticas alimentares não saudáveis. Em 2017, a alimentação inadequada foi o principal fator de risco global para mortalidade e o segundo principal para anos de vida perdidos por incapacidade^1^ . Observa-se, também, o seu efeito no sistema alimentar de um país. No Brasil, o aumento na produção e disponibilidade de alimentos ultraprocessados tem influenciado o consumo e a saúde da população^2^ .

Com base nisso, em 2014, o Ministério da Saúde publicou a segunda edição do *Guia Alimentar para População Brasileira* , que traz princípios e recomendações para uma alimentação saudável e sustentável. As recomendações do Guia consideram a alimentação mais do que um meio de obtenção de nutrientes, incorporando mensagens que levam em conta o processamento de alimentos, as combinações na forma de refeições, as maneiras de comer, bem como o impacto sobre o meio ambiente e a sociedade^3^ .

Ainda é um desafio avaliar e monitorar a alimentação considerando o Guia^3^ , devido à ausência de instrumentos. A alimentação tem sido avaliada com aplicação de índices de qualidade da dieta baseados em recomendações anteriores, que dependem do uso de inquéritos alimentares e demandam tempo para análise^4^ . Além disso, esses índices se sustentam em uma definição subjetiva de pontos de corte de classificação, conferindo o mesmo peso para itens com diferentes impactos na saúde. A teoria de resposta ao item (TRI) é uma proposta de análise para superar essa limitação ao considerar as características dos itens do questionário quanto à capacidade de discriminar a variável de interesse e localização no respectivo *continuum* e um modelo probabilístico para calcular e descrever os escores^5^ .

Diante desta lacuna, a escala de qualidade da dieta (ESQUADA) foi desenvolvida com base nas recomendações do Guia^3^ , contemplando, por exemplo, costume de realizar refeições principais e consumir alimentos como frutas e bebidas açucaradas^6^ . O presente estudo buscou avaliar duas evidências de validade da ESQUADA para seleção dos itens com melhor discriminação da qualidade da dieta e propor uma descrição em níveis de escores.

## MÉTODOS

Este é um estudo psicométrico para avaliar a validade da ESQUADA quanto à estrutura interna e comportamento diferencial, identificando o conjunto final de itens, com aplicação da TRI.

Em estudo anterior^6^ , especialistas foram convidados a avaliar os itens quanto à pertinência para medir a qualidade da dieta, à coerência com as recomendações do Guia^3^ e à clareza da escrita para adolescentes e jovens adultos^7^ . Após analisar as sugestões dos especialistas, foi avaliada a compreensão dos itens por adolescentes e adultos brasileiros^6^ . Posteriormente, foi considerado um total de 52 itens para as análises da estrutura interna e comportamento diferencial, apresentadas no presente estudo, que, para melhor organização, foi dividido em duas fases, descritas a seguir ( Figura 1 ).

**Figura 1:** Descrição das fases do estudo de duas evidências de validade da escala de qualidade da dieta (ESQUADA).

TRI: teoria de resposta ao item; ESQUADA: escala de qualidade da dieta.

### Coleta de Dados para o Estudo de Validação

A amostra de respondentes buscou ser representativa do traço latente em estudo^8^ (qualidade da dieta), incluindo indivíduos dos seus diferentes níveis.

Para minimizar o desgaste ao responder o questionário, os itens foram organizados em treze formulários com aplicação do desenho de blocos incompletos balanceados^6 , 9^ . Cada formulário era composto por dezesseis itens sobre qualidade da dieta e outros sobre características sociodemográficas, como data de nascimento, sexo, nível de educação e país (de nascimento e residência atual).

A ordem de apresentação dos itens nos formulários também foi alterada para minimizar a influência da posição e do desgaste na qualidade das respostas. Os formulários foram inseridos na plataforma *Survey Monkey* e numerados em ordem crescente de um a treze. O acesso foi realizado por meio de um endereço eletrônico com um sistema de programação para garantir a apresentação sequencial dos questionários, com o objetivo de garantir uma distribuição igualitária de respondentes por formulário. Esse endereço eletrônico foi compartilhado em redes sociais, listas de e-mail e rede de comunicação interna da Universidade de São Paulo.

Durante a coleta, foi avaliada a necessidade de direcionar a aplicação dos formulários para indivíduos com determinado nível de qualidade da dieta ainda pouco representado. Para atender a essa representatividade do traço latente, foi feita uma coleta complementar com alunos do ensino médio e educação para jovens adultos de escolas públicas do estado de São Paulo, mediante autorização da Secretaria Estadual, utilizando o laboratório de informática e com a presença de um pesquisador.

A coleta de dados foi realizada entre março e abril de 2018, incluindo adolescentes e adultos brasileiros, com idade entre 15 e 50 anos, residentes no Brasil. Todos os indivíduos assinaram um termo de consentimento livre e esclarecido on-line, registrando o desejo em participar. O estudo foi aprovado pelo Comitê de Ética para Pesquisa com Seres Humanos da Faculdade de Saúde Pública (protocolo número 1.943.099).

Dentre os indivíduos que acessaram os formulários (n = 2.373), foram excluídos aqueles que não atenderam aos critérios de inclusão (n = 270), não responderam ou responderam a apenas um dos itens sobre qualidade da dieta (n = 11) e aqueles que acessaram os formulários mais de uma vez (n = 33). A amostra final incluiu 2.059 indivíduos.

### Análise dos Dados para Construção da Escala

As categorias de respostas foram numeradas em ordem crescente a partir do número 1, em coerência com a característica acumulativa do traço latente. Nesse sentido, o número 1 identificou a opção de resposta cujo costume sugere uma pior qualidade da dieta. Por exemplo, o item sobre substituir a refeição do almoço ou jantar por lanches apresentava cinco opções de resposta: “Não costumo”, “Sim, às vezes”, “Sim, eu costumo substituir em um ou dois dias na semana”, “Sim, eu costumo substituir em três ou quatro dias na semana” e “Sim, eu costumo substituir em cinco ou mais dias”. Enquanto a maior frequência de substituição, que sugere pior qualidade da dieta, foi identificada com o número 1; o não costume foi identificado com o número 5.

#### Análise da estrutura interna do conjunto de itens

O estudo da dimensionalidade foi realizado com a análise fatorial de informação completa, considerando unidimensionalidade quando o primeiro fator explica 20% ou mais da variância^10^ . Itens com comunalidade menor do que 0,20 e carga fatorial inferior a 0,30^11^ foram excluídos das análises seguintes.

Na sequência, a probabilidade dos indivíduos escolherem a categoria k para cada item foi calculada com aplicação do modelo de resposta gradual de Samejima, representado pela equação (1)^12^ :


$$P_{i,k}\left(\theta_{j}\right)=\frac{1}{1+\exp^{-a_{i}\left(\theta_{j}-b_{i,k}\right)}}-\frac{1}{1+\exp^{-a_{i}\left(\theta_{j}-b_{i,k+1}\right)}}$$ (1)

onde:

θ_j_– escore da TRI para o indivíduo *j* ;

P_i,k_(θ_j_) – probabilidade do indivíduo *j* escolher a categoria *k* ao responder o item *i* ;

a_i_– parâmetro de discriminação do item *i* ;

b_i,k_– parâmetro de localização da categoria *k* do item *i* , com b_i,2_≤ b_i,3_≤ b_i,4_≤ ….

O parâmetro de discriminação (a_i_) indica a capacidade de cada item de discriminar indivíduos com diferentes níveis do traço latente. Itens com maiores valores desse parâmetro apresentam melhor discriminação do traço latente^5^ . Itens com baixa discriminação, ou seja, com parâmetro a_i_igual ou inferior a 0,70, foram excluídos das análises^8^ .

O parâmetro de localização (b_i,k_) identifica a localização das categorias de resposta de cada item em todo o *continuum* do traço latente^5^ . Itens com estimativas de b_i,k_com altos erros-padrões, quando comparados com outros itens^13^ , e com sobreposição na curva característica do item (CCI), a representação gráfica do modelo acima, foram recategorizados. Aqueles itens que mantiveram altos valores de erro-padrão mesmo após recategorização foram excluídos das análises.

O ajuste do modelo foi avaliado pelos índices *root mean square error of approximation* (RMSEA), *standardized root mean square residual* (SRMSR), *comparative fit index* (CFI) e *Tucker–Lewis index* (TLI)^14 , 15^ . A qualidade do ajuste é confirmada quando valores de RMSEA e SRMSR são menores ou iguais a 0,05 e valores de CFI e TLI são maiores do que 0,90^16^ . A acurácia da ESQUADA também foi analisada a partir da curva de informação do teste (CIT), a qual representa a soma das informações dos itens que a compõem. O estudo da confiabilidade empírica, uma medida análoga ao α de Cronbach, permitiu identificar a confiabilidade da ESQUADA, aceita quando valor superior a 0,70^11^ .

#### Análise da consequência do uso

Considerando que idade e sexo podem influenciar a probabilidade do indivíduo *j* escolher a categoria *k* para o item *i* , o comportamento diferencial do item ( *differential item functioning* – DIF) foi avaliado segundo essas variáveis. O valor de p menor do que 0,05 foi adotado como nível de significância. A detecção do DIF também foi avaliada em análise da CIT^17^ .

#### Construção da escala

A probabilidade em escolher a categoria *k* foi calculada para cada item em diferentes escores da qualidade da dieta. O item foi posicionado no escore em que essa probabilidade foi maior ou igual a 0,5. Itens com melhor discriminação da qualidade da dieta (a_i_≥ 1,00) foram considerados âncoras para descrição das práticas alimentares e consumo de alimentos característicos de cada escore. Os escores foram agrupados em níveis indicativos da característica acumulativa da qualidade da dieta, permitindo identificar que quanto maior o escore, menor a frequência de substituir refeições por lanches, por exemplo. Essa etapa foi realizada por quatro nutricionistas (TSSS, MLCL, MAAA e BSV). Para melhor interpretabilidade da ESQUADA^5^ , os escores estimados em uma escala com média igual a 0 e desvio-padrão igual a 1 foram, posteriormente, transformados para uma escala com média igual a 250 e desvio-padrão igual a 50.

As análises foram realizadas no software R (pacotes *crossdes* e *mirt* ) e no Microsoft Excel versão 2013.

## RESULTADOS

Os respondentes representaram a variabilidade do traço latente de modo a garantir a estimação dos parâmetros a_i_e b_i,k_. A maioria desses respondentes era do sexo feminino (70%), não tinha ensino superior (58%) e vivia na região Sudeste do Brasil (79%). O preenchimento dos formulários demandou um tempo entre seis e oito minutos.

Os 52 itens (I-01 a I-52) considerados para a análise explicaram 0,20 da variância, mas 15 deles foram excluídos devido aos baixos valores de comunalidade, carga fatorial e parâmetro de discriminação (I-06, I-17, I-18, I-19, I-20, I-23, I-26, I-27, I-28, I-30, I-33, I-34, I-38, I-45 e I-49). As análises foram repetidas sem esses itens (n = 37), recategorizando aqueles com estimativas com altos valores de erro-padrão para o parâmetro b_i,k_e sobreposição na CCI. Nas análises posteriores, 12 desses itens apresentaram baixos valores de comunalidade, carga fatorial e parâmetro de discriminação e foram excluídos (I-05, I-07, I-10, I-12, I-14, I-15, I-16, I-29, I-35, I-37, I-39 e I-47).

Os 25 itens restantes apresentaram valores adequados dos parâmetros a_i_e b_i,k_e respectivos erros-padrões, explicando 0,34 da variância. Os valores de comunalidade, carga fatorial e parâmetros a_i_e b_i,k_estão descritos na Tabela 1 . Os próximos resultados se concentram nos 25 itens que permaneceram no conjunto final, compondo a ESQUADA. Itens e opções de resposta estão apresentados na Figura 2 .

**Tabela 1:** Descrição dos valores de comunalidade, carga fatorial e parâmetros de discriminação e localização e respectivos erros padrões de cada item da escala de qualidade da dieta (ESQUADA) (n = 2.059).

Itens	Comunalidade	Carga fatorial	a_i_(EP)	b_i,2_(EP)	b_i,3_(EP)	b_i,4_(EP)
I-01-Você costuma tomar café da manhã?	0,364	0,603	1,288 (0,154)	-0,637 (0,090)	-	-
I-02-Quais alimentos você costuma comer no café da manhã?	0,241	0,491	0,959 (0,125)	-1,673 (0,198)	-0,108 (0,098)	-
I-03-Você costuma almoçar?	0,264	0,514	1,021 (0,202)	-2,750 (0,427)	-	-
I-04-Quais alimentos você costuma comer no almoço?	0,372	0,610	1,309 (0,343)	-3,457 (0,662)	-	-
I-08-Você costuma substituir a refeição do almoço ou jantar por lanches? (Considere exemplos de lanches: pizza, salgados, Hot Hit^®^, Hot Pocket^®^, X-salada, X-ovo, Mc Donald’s^®^, Bob’s^®^, Subway^®^, escondidinho industrializado, estrogonofe industrializado ou lasanha industrializada.).	0,264	0,514	1,020 (0,114)	-3,429 (0,356)	-1,937 (0,195)	0,521 (0,101)
I-09-Você costuma cozinhar ou ajudar no preparo dos alimentos em refeições como almoço e jantar? (Considere ajudar no preparo: lavar, picar e/ou cozinhar os alimentos.).	0,206	0,454	0,868 (0,132)	-0,245 (0,111)	-	-
I-11-Onde você ou alguém da sua casa costuma adquirir frutas, legumes e/ou verduras?	0,176	0,419	0,786 (0,107)	0,304 (0,117)	2,305 (0,294)	-
I-13- “Por mais R$1,00 leve a porção grande de batata frita”. Em situações como a do exemplo apresentado, você costuma escolher a maior porção dos alimentos pagando um pouco mais?	0,239	0,489	0,954 (0,127)	0,457 (0,108)	-	-
I-21-Você costuma comer arroz integral e/ou macarrão integral?	0,261	0,511	1,011 (0,110)	-0,029 (0,095)	1,278 (0,145)	2,932 (0,298)
I-22-Você costuma comer aveia, quinoa e/ou centeio?	0,476	0,690	1,624 (0,163)	-0,027 (0,068)	1,291 (0,106)	2,494 (0,196)
I-24-Você costuma comer legumes ou verduras crus e/ou cozidos? (Não considerar o consumo de mandioca, batata, cará e inhame.).	0,414	0,643	1,429 (0,147)	-2,069 (0,176)	-0,771 (0,089)	0,599 (0,087)
I-25-Você costuma comer frutas? (Não considerar o consumo de sucos.).	0,295	0,544	1,102 (0,118)	-2,622 (0,254)	-0,768 (0,105)	0,888 (0,117)
I-31-Você costuma comer castanha do Pará/Brasil, castanha de caju e/ou nozes?	0,362	0,601	1,281 (0,139)	-0,409 (0,084)	1,075 (0,114)	3,080 (0,291)
I-32-O que você costuma beber quando está com sede?	0,200	0,448	0,852 (0,171)	-2,743 (0,475)	-	-
I-36-Você costuma comer bolos, bolachas ou biscoitos industrializados (comprados prontos)? (Considerar também aqueles feitos com massas prontas.).	0,276	0,525	1,050 (0,117)	-0,863 (0,118)	1,601 (0,164)	-
I-40-Você costuma comer ketchup, mostarda e/ou maionese industrializados (comprados prontos)?	0,199	0,446	0,849 (0,117)	-2,249 (0,286)	1,759 (0,236)	-
I-41-Você costuma comer lanches como salgados fritos ou assados, hambúrguer tipo fast-food, cachorro-quente e/ou pizza industrializada (comprada pronta)? (Considerar como exemplos de hambúrguer fast-food: X-salada, X-ovo, Hot Hit…).	0,429	0,655	1,476 (0,153)	-2,853 (0,248)	-1,286 (0,111)	1,339 (0,118)
I-42-Você costuma comer cereais matinais e/ou barrinhas de cereais industrializados? (Considerar como exemplos de cereais matinais: Sucrilhos^®^, Nescau Cereal^®^…).	0,165	0,407	0,758 (0,127)	-3,738 (0,582)	-0,824 (0,164)	-
I-43- Você costuma comer salgadinhos de pacote (tipo chips) como: Ruffles^®^, Cheetos^®^, Elma Chips^®^, Doritos^®^, Pringles^®^, ou pipoca de micro-ondas?	0,448	0,670	1,535 (0,158)	-3,117 (0,287)	-1,979 (0,164)	0,439 (0,073)
I-44-Você costuma beber refrigerantes e/ou sucos em pó, de caixinha, em lata e/ou garrafa? (Considerar como exemplos: Del Valle^®^, Maguary^®^…).	0,514	0,717	1,749 (0,162)	-0,430 (0,071)	0,863 (0,084)	-
I-46-Você costuma comer caldas/coberturas industrializadas para sorvete, geleias industrializadas, doce de leite…?	0,317	0,563	1,160 (0,133)	-1,891 (0,188)	1,118 (0,133)	-
I-48-Você costuma beber bebidas lácteas como Toddynho^®^?	0,494	0,703	1,682 (0,187)	-1,608 (0,133)	-0,156 (0,068)	-
I-50-Você costuma comer mortadela, salame, patês/pastas industrializados com sabor de carne, peito de peru/frango…?	0,344	0,586	1,231 (0,123)	-1,607 (0,149)	0,906 (0,107)	-
I-51-Você costuma comer *nuggets/steak* (frango empanado industrializado), salsicha e/ou hambúrguer industrializado (comprado pronto)?	0,558	0,747	1,912 (0,199)	-2,471 (0,191)	-1,560 (0,113)	0,319 (0,066)
I-52-Quando você está em casa, você costuma comer macarrão instantâneo (miojo), sopas em pó, alimentos/pratos congelados industrializados e/ou hambúrguer tipo fast-food? (Considerar como exemplos: Nissin^®^…).	0,633	0,796	2,237 (0,212)	-2,292 (0,158)	-1,473 (0,097)	0,134 (0,060)

EP: erro-padrão; a_i_: parâmetro de discriminação do item *i* ; b_i,2_: parâmetro de locação da categoria 2 do item *i* ; b_i,3_: parâmetro de locação da categoria 3 do item *i* ; b_i,4_: parâmetro de locação da categoria 4 do item *i* .

**Figura 2:** Questionário com itens e opções de resposta que compõem a escala de qualidade da dieta (ESQUADA).

O item sobre os locais onde os indivíduos costumam comprar frutas e hortaliças apresentou a menor discriminação da qualidade da dieta (a_11_= 0,786). Por outro lado, o item sobre o costume de comer alimentos ultraprocessados em casa apresentou a melhor discriminação (a_52_= 2,237).

O item sobre o costume de comer cereais matinais e/ou barrinhas de cereais apresentou o menor valor para o parâmetro de localização (b_42,2_= -3,738), sugerindo que esses alimentos são consumidos com maior frequência por indivíduos com menores níveis de qualidade da dieta. Em contrapartida, os itens sobre o costume de comer castanhas e/ou nozes (b_31,4_= 3,080) e arroz/macarrão integral (b_21,4_= 2,932) apresentaram os maiores valores de b_i,k_, sugerindo que esses alimentos são consumidos mais frequentemente por indivíduos com maiores níveis de qualidade da dieta.

A ESQUADA apresentou um excelente ajuste (RMSEA = 0,01; SRMSR = 0,02; CIF = 0,99 e TLI = 0,99), com melhor acurácia entre os escores -2 e +2 ( Figura 3 ) e uma adequada confiabilidade empírica (0,70). Não foi identificado comportamento diferencial para nenhum item, considerando tanto idade quanto sexo.

**Figura 3:** Curva de informação da escala de qualidade da dieta (ESQUADA) (n = 2.059).

θ: escore; I(θ): Informação do escore.

Para melhor interpretabilidade da ESQUADA, a transformação dos escores para escala com média igual a 250 e desvio-padrão igual a 50 foi obtida pela constante: θ_(250,50)_= 59,09 × θ_(0,1)_+ 250,12. Os escores foram agrupados para apresentar os cinco níveis de interpretação da ESQUADA: “muito ruim”, “ruim”, “boa”, “muito boa” e “excelente” ( Quadro 1 ). Os itens e respectivas opções de respostas que compõem cada nível estão indicados na Figura 4 .

**Quadro 1:** Breve descrição dos níveis da escala de qualidade da dieta (ESQUADA).

Classificação	Escala (0,1)	Escala (250,50)	Breve descrição
Muito ruim	Menor ou igual a -2	Menor ou igual a 150	Indivíduos consomem ultraprocessados e substituem refeições por lanches em um ou dois dias na semana. Indivíduos consomem frutas, legumes e verduras em menos do que um dia na semana.
Ruim	Maior do que -2 e menor ou igual a -1	Maior do que 150 e menor ou igual a 200	Indivíduos mantêm o consumo de frutas, legumes e verduras em menos do que um dia na semana. Indivíduos consomem ultraprocessados e substituem refeições por lanches em menos do que um dia na semana.
Boa	Maior do que -1 e menor ou igual a 0,5	Maior do que 200 e menor ou igual a 275	Indivíduos mantêm a substituição de refeições por lanches em menos do que um dia na semana. Indivíduos não consomem alguns ultraprocessados, porém consomem bebidas açucaradas em menos do que um dia na semana. Indivíduos consomem frutas, legumes e verduras de um a quatro dias na semana e aveia, centeio, quinoa, castanhas, nozes e arroz/macarrão integral em menos do que um dia na semana. Indivíduos tomam o café da manhã em pelo menos um dia na semana.
Muito boa	Maior do que 0,5 e menor ou igual a 2,5	Maior do que 275 e menor ou igual a 375	Indivíduos não substituem refeições por lanches. Indivíduos não consomem nenhum ultraprocessado. Indivíduos mantêm a realização do café da manhã em pelo menos um dia na semana. Indivíduos consomem frutas, legumes e verduras em cinco ou mais dias na semana e aveia, centeio, quinoa, castanhas, nozes e arroz/macarrão integral de um a quatro dias na semana.
Excelente	Maior do que 2,5	Maior do que 375	Indivíduos mantêm a não substituição de refeições por lanches e o não consumo de alimentos ultraprocessados. Indivíduos mantêm a realização do café da manhã em pelo menos um dia na semana e o consumo de frutas, legumes e verduras em cinco ou mais dias na semana. Indivíduos consomem aveia, centeio, quinoa, castanhas, nozes e arroz/macarrão integral em cinco ou mais dias na semana.

**Figura 4:** Posicionamento dos itens e respectivas opções de resposta nos cinco níveis da escala de qualidade da dieta (ESQUADA).

b_i,k_: parâmetro de localização da categoria *k* do item *i* .

## DISCUSSÃO

Neste estudo, foram avaliadas duas evidências de validade da ESQUADA, escala de qualidade da dieta que engloba consumo de alimentos in natura e minimamente processados e ultraprocessados e práticas alimentares (como tomar café da manhã, cozinhar e substituir refeições por lanches). O uso da TRI permitiu selecionar itens com melhor discriminação da qualidade da dieta e os resultados das análises do ajuste do modelo e confiabilidade sustentam a acurácia da medida apresentada pela ESQUADA. A CIT permitiu visualizar o erro da medida em toda a escala, sugerindo que a ESQUADA apresenta melhor acurácia entre os escores -2 e +2. Além disso, a análise de comportamento diferencial do item indicou que a medida é válida em diferentes amostras. Ou seja, a ESQUADA mede com a mesma precisão a qualidade da dieta de indivíduos independentemente do sexo e da idade. A ausência de DIF destaca o estudo prévio quanto à clareza dos itens para adolescentes e adultos brasileiros^6^ . As análises também permitiram a proposta de descrição das práticas alimentares e consumo de alimentos em cinco níveis.

Após a publicação do Guia^3^ , evidenciaram-se três aspectos: (1) a compreensão de que alimentos não são apenas fontes de nutrientes; (2) a importância de considerar não apenas o consumo de alimentos, mas também as práticas alimentares; e (3) as limitações metodológicas da estatística utilizada no desenvolvimento dos índices de qualidade da dieta. A ESQUADA tem como objetivo avaliar a qualidade da dieta em coerência com as recomendações do Guia^3^ , considerando outras características da alimentação que não apenas o fator nutricional, com aplicação de uma estatística mais robusta. A ESQUADA se apresenta, então, como a primeira escala que avalia a qualidade da dieta em coerência com o Guia^3^ , com aplicação da TRI, uma análise centrada nos itens e não apenas no escore.

Os estudos no campo da Nutrição expressam de forma hegemônica o paradigma biomédico à luz da lógica das ciências naturais, limitando a compreensão das múltiplas formas como a alimentação interfere na vida das pessoas. O significado de alimentar-se vem sofrendo mudanças, isto é, de um conceito reducionista, no sentido de fornecimento exclusivo de energia e nutrientes, passa a expressar as experiências do ser humano, marcadas pela natureza e pela cultura. Nesse sentido, o comer é um ato natural, que se expressa das formas mais culturais: nos modos de comer e preparar os alimentos, nas escolhas e preferências alimentares, na comensalidade e em todas as transformações do alimento até o consumo^20 , 21^ .

Na ESQUADA, o costume de cozinhar em pelo menos um dia na semana, avaliado pelo I-09, identificou uma melhor qualidade da dieta, indicando o cuidado em reservar algum tempo para cozinhar durante a semana, mesmo com a intensa rotina de trabalho. Considerando que a população está cozinhando em casa com menor frequência^22^ , embora um dia por semana seja ainda insuficiente, o item consegue discriminar a qualidade da dieta.

Os itens sobre consumo de hortaliças (I-24) e frutas (I-25) e local de compra desses alimentos (I-11) aderiram aos critérios para manutenção no conjunto final. O consumo de hortaliças é ponto relevante para alimentação saudável e estratégia global para prevenção e controle das doenças crônicas não transmissíveis^23^ . O Guia recomenda preferir o consumo de frutas a beber suco natural, bem como comprar alimentos em locais onde exista maior disponibilidade de alimentos in natura^3^ . O local onde a população se abastece de frutas e hortaliças também pode indicar a qualidade da dieta. No Brasil, as compras em feiras de ruas, pequenos mercados ou diretamente com o produtor relacionaram-se com menor consumo de alimentos ultraprocessados^24^ .

Um dos itens da ESQUADA (I-32) buscou investigar a bebida ingerida pelos indivíduos quando estão com sede: água, suco de fruta natural ou bebidas açucaradas. Ele apresentou uma discriminação adequada da qualidade da dieta, com baixos valores para o parâmetro de localização, ou seja: indivíduos com baixos escores de qualidade da dieta ingerem água quando estão com sede. Em acordo com as recomendações para indicadores de qualidade da dieta^25^ , a ESQUADA não quantifica a ingestão de água em mililitros ou copos, porém avalia um costume diretamente relacionado com alimentação saudável e inversamente com incidência de obesidade^26^ . A presença desse item na ESQUADA destaca sua importância na avaliação da qualidade da dieta.

Evidências científicas apontam a importância do café da manhã para nutrição e saúde^27^ . A descrição da ESQUADA ( Quadro 1 ) indica que a qualidade da dieta aumenta à medida que os indivíduos adotam o costume de tomar café da manhã, almoçar e não substituir refeições por lanches. Deve-se considerar que alimentação é um comportamento complexo. Por exemplo, em um nível de qualidade da dieta “muito ruim”, os indivíduos costumam almoçar uma refeição tradicional (com arroz, feijão, carne ou opção vegetariana e/ou salada) e substituir refeições por lanches e consumir alimentos ultraprocessados. Por outro lado, o costume de tomar café da manhã só foi observado a partir do nível de “boa” qualidade da dieta, no qual há uma menor frequência de substituição de refeições por lanches e consumo de alimentos ultraprocessados. Ou seja, o costume de tomar café da manhã está presente em um nível de qualidade da dieta em que os indivíduos já costumam almoçar e consomem ultraprocessados com menor frequência.

Dentre os 25 itens da ESQUADA, 11 avaliam o consumo de alimentos ultraprocessados. O item que investiga o consumo de cereais matinais e barrinhas de cereais (I-42) apresentou o menor valor para o parâmetro de localização (b_42,2_= -3,738), sugerindo que esses alimentos são consumidos frequentemente nos níveis mais baixos de qualidade da dieta. O consumo frequente de alimentos ultraprocessados associou-se a um pior perfil nutricional em adolescentes e adultos^28^ . Esse consumo tem sido estimulado pelas estratégias de marketing, como uso de termos nos rótulos de alimentos, por exemplo: tradicional e caseiro^29^ . Contemplando um pouco dessa influência, a ESQUADA inclui um item que avalia o costume de escolher a maior porção de alimentos quando a diferença de preço é pequena em relação à menor porção (I-13).

Na descrição da ESQUADA ( Quadro 1 ), observa-se que os indivíduos deixam de consumir alguns alimentos ultraprocessados no mesmo nível de qualidade da dieta que começam a consumir cereais integrais (I-21, I-22) e oleaginosas (I-31). Os ultraprocessados só deixam de fazer parte da alimentação nos últimos dois níveis da ESQUADA, quando também se observa que alimentos in natura e minimamente processados estão incorporados na alimentação. Esses resultados reforçam a complexidade de avaliar a qualidade da dieta. Assim, indivíduos com escores medianos, ou seja, com “boa” qualidade da dieta, apresentam práticas alimentares saudáveis e não saudáveis concomitantemente. Diante dessa complexidade, a aplicação da TRI permitiu identificar os itens que melhor discriminam a qualidade da dieta^5^ .

Portanto, a ESQUADA é apresentada como uma nova medida para avaliar com acurácia a qualidade da dieta nos próximos estudos. Para isso, os parâmetros de discriminação e localização e a constante de transformação aqui apresentados deverão ser considerados nas próximas aplicações da ESQUADA. Com a técnica de equalização^5^ , pesquisadores poderão incluir outros itens na medida, estimando seus parâmetros com base na discriminação e localização daqueles que compõem a ESQUADA e ampliando a avaliação da qualidade da dieta. Além disso, A ESQUADA é de fácil aplicação e análise e, com sua reaplicação, é possível avaliar os escores de um mesmo grupo de indivíduos, por exemplo, após uma intervenção em saúde e nutrição.

Alguns aspectos positivos e limitações deste estudo devem ser apontados. Mesmo que a ESQUADA avalie diferentes aspectos da qualidade da dieta, o conjunto final de itens está mais relacionado a questões relativas ao consumo de alimentos. Além disso, as análises indicaram a necessidade de recategorização de algumas opções de resposta devido ao menor número de indivíduos que as selecionaram como resposta. Entretanto, o número de respondentes foi suficiente para obter estimativas adequadas dos parâmetros dos itens, bem como uma informação acurada da qualidade da dieta. O estudo apresenta destaques: a ESQUADA se baseia nas atuais recomendações para alimentação da população brasileira e em análises mais robustas para seleção dos itens com melhor discriminação da qualidade da dieta, permitindo localizar e descrever consumo e práticas alimentares no seu *continuum* .

## CONCLUSÃO

A ESQUADA é composta por 25 itens que avaliam consumo e práticas alimentares em concordância com o Guia. As exclusões não contrariam as recomendações vigentes, permitindo selecionar os itens mais precisos para avaliar a qualidade da dieta nos próximos estudos e contribuir para o estado da arte da epidemiologia nutricional.

Nesse sentido, itens sobre o costume de cozinhar, tomar café da manhã, local onde os indivíduos compram frutas e hortaliças e consumo de alimentos in natura e ultraprocessados exemplificam o conjunto final que compõe a ESQUADA. As análises também providenciaram um detalhamento do consumo e práticas alimentares característicos de cada nível de qualidade da dieta. De maneira a complementar o estudo das duas evidências de validade da ESQUADA apresentadas, futuros trabalhos poderão investigar as associações entre os escores e outras medidas relacionadas com saúde e nutrição.
