# Supplementary material for: Erratum
Source: Rev Saude Publica. 2023 Mar 15;57:11. doi: 10.11606/s1518-8787.2023057002397err (PMC10118419; doi:10.11606/s1518-8787.2023057002397err)
Supplement: Translation

# Translation: Errata

No artigo “ **Duas evidências de validade da ESQUADA e níveis de qualidade da dieta dos brasileiros** ”, DOI https://doi.org/10.11606/s1518-8787.2021055002397, publicado na Revista de Saúde Pública.2021;55:39, na página 10, a RSP corrige um trecho do último parágrafo da seção resultados.

**Onde se lê:**

θ_(250,50)_ = 59,09 × θ_(0,1)_ + 250.12

**Leia-se:**

θ_(250,50)_ = 50 × θ_(0,1)_ + 250
